# Can urologists introduce the concept of “oligometastasis” for metastatic bladder cancer after total cystectomy?

**DOI:** 10.18632/oncotarget.22911

**Published:** 2017-12-04

**Authors:** Koichiro Ogihara, Eiji Kikuchi, Keitaro Watanabe, Ryohei Kufukihara, Yoshinori Yanai, Kimiharu Takamatsu, Kazuhiro Matsumoto, Satoshi Hara, Masafumi Oyama, Tetsuo Monma, Takeshi Masuda, Shintaro Hasegawa, Mototsugu Oya

**Affiliations:** ^1^ Department of Urology, Keio University School of Medicine, Tokyo, Japan; ^2^ Department of Urology, National Hospital Organization Tochigi Medical Center, Tochigi, Japan; ^3^ Department of Urology, National Hospital Organization Saitama Hospital, Saitama, Japan; ^4^ Department of Urology, Saitama City Hospital, Saitama, Japan; ^5^ Department of Uro-Oncology, Saitama Medical University International Medical Center, Saitama, Japan; ^6^ Department of Urology, Kawasaki Municipal Hospital, Kanagawa, Japan

**Keywords:** metastatic bladder cancer, total cystectomy, oligometastasis, prognosis, chemotherapy

## Abstract

We investigated whether the concept of oligometastasis may be introduced to the clinical management of metastatic bladder cancer patients. Our study population comprised 128 patients diagnosed with metastatic bladder cancer after total cystectomy at our 6 institutions between 2004 and 2014. We extracted independent predictors for identifying a favorable. Occurrence that fulfilled all 4 criteria which were independently associated with cancer-specific death was defined as oligometastasis: a solitary metastatic organ; number of metastatic lesions of 3 or less; the largest diameter of metastatic foci of 5cm or less; and no liver metastasis. We evaluated differences in clinical outcomes between patients with oligometastasis (oligometastasis group) and those without oligometastasis (non-oligometastasis group). Overall, there were 43 patients in the oligometastasis group. The 2-year cancer-specific survival rate in the oligometastasis group was 53.3%, which was significantly higher than that in the non-oligometastasis group (16.1%, p<0.001). A multivariate analysis revealed that non-oligometastasis (p<0.001), not performing salvage chemotherapy (p<0.001), and not performing metastatectomy (p=0.028) were independent risk factors for cancer-specific death. In the subgroup of 83 patients who received salvage chemotherapy, 30 were in the oligometastasis group. The 2-year cancer-specific survival rate in the oligometastasis group was 55.0%, which was significantly higher than that in the non-oligometastasis group (22.0%, p=0.005). Non-oligometastasis (p=0.009) was the only independent risk factor for cancer-specific death. We presented that urothelial carcinoma with oligometastasis had a favorable prognosis and responded to systemic chemotherapy. Oligometastasis may be treated as a separate entity in the field of metastatic urothelial carcinoma.

## INTRODUCTION

Among various types of malignant tumors, the prognosis of metastatic bladder cancer (BC) is known to be poor, and its treatment mainly involves systemic chemotherapy. Combinations of the methotrexate, vinblastine, doxorubicin, and cisplatin (MVAC) or gemcitabine and cisplatin (GC) regimen have been established as chemotherapy for metastatic BC [1, 2], and the initial response rate of metastatic BC to chemotherapy and median overall survival were previously reported to be approximately 60% and 13-14 months, respectively [1]. Although we have encountered long-term survivors with metastatic BC who lived for more than 5 years after the diagnosis of metastasis, the characteristics of this type of metastatic BC have not yet been investigated in detail.

The concept of oligometastasis was recently introduced for various cancers, such as breast [3], lung [4], and prostate cancers [5]. The theory of oligometastasis was first proposed as a sequel to the spectrum theory of cancer metastasis by Hellman et al. in 1995 [6]. In oligometastatic disease, cancer cells are sloughed out of the primary tumor and land at target organs. However, since they do not have the properties necessary to survive the circulation and invade target organs, the prognosis of patients with oligometastasis appears to be good. Oligometastasis is defined as a state of metastatic disease that is limited in total disease burden, typically by the number of clinically evident or radiographic sites, and does not rapidly spread to more sites [7]. Mertens et al. have already suggested the concept of oligometastasis in BC [8]. They mentioned that the optimal imaging modalities might be important for the definition of oligometastatic BC and further study evaluating detailed clinical features of defined oligometastasis in metastatic BC would be warranted. Furthermore, so far there is no clear criteria for defined oligometastasis in the field of urothelial carcinoma (UC). Therefore, we proposed to firstly define oligometastasis and determine whether the concept of defined oligometastasis could have an impact on clinical outcome in metastatic BC.

We herein focused on oligometastasis and investigated whether the concept of oligometastasis may be introduced to the clinical management of metastatic BC patients, particularly those receiving salvage chemotherapy. The clinical questions of our study were 1) Is it possible to introduce the concept of oligometastasis to metastatic BC after total cystectomy (TC)? and 2) Whether the oligometastasis group has a better prognosis among patients receiving salvage chemotherapy?.

## RESULTS

### The definition of oligometastasis and patients stratified

As shown in Table 1, we found that four indicators: the number of metastatic organs (solitary vs. multiple organs), number of metastatic lesions (≤3 vs. 3<), the largest diameter in metastatic foci (≤5cm vs. 5cm<), and presence or absence of liver metastasis in addition to salvage chemotherapy, were independently associated with cancer-specific death. Some of these four indicators: the number of metastatic organs, number of metastatic lesions, the largest diameter in metastatic foci, and metastatic organ sites, have frequently been utilized to define oligometastasis in other types of cancers [9-11]. Therefore, in the present study, we defined oligometastasis as having “a solitary metastatic organ, number of metastatic lesions of ≤3, the largest diameter of metastatic foci of ≤5cm, and absence of liver metastasis. We evaluated differences in clinical outcomes between patients with oligometastasis (oligometastasis group; OM group) and those without oligometastasis (non-oligometastasis group; non-OM group).

**Table 1:** Uni- and multivariate analysis for cancer-specific death according to clinicopathological features including the metastatic number, tumor diameter, and tumor site in overall patients

	Univariate	Multivariate	
	p value	HR (95% CI)	p value
Sex	0.947		
Male			
Female			
Age at diagnosis	0.388		
<70			
70≤			
Previous history of smoking	0.305		
No			
Yes			
PS at diagnosis of metastasis	0.001		0.102
≤1		1	
2≤		1.77 (0.89-3.5)	
Clinical T stage	0.352		
<cT3			
cT3≤			
Clinical N stage	0.094		
cN0			
cN1≤			
Number of lymph nodes removed at TC	0.119		
<10			
10≤			
Pathological T stage	0.049		0.713
<pT3		1	
pT3≤		1.09 (0.68-1.76)	
Pathological N stage	0.472		
pN0			
pN1≤			
Histological type	0.734		
Pure UC			
Non-pure UC			
Concomitant CIS on TC specimens	0.541		
No			
Yes			
LVI status on TC specimens	0.005		0.409
Negative		1	
Positive		1.23 (0.75-2.01)	
Neoadjuvant chemotherapy	0.612		
Yes			
No			
Adjuvant chemotherapy	0.45		
Yes			
No			
Salvage chemotherapy	0.001		0.008
Yes		1	
No		2.12 (1.22-3.68)	
Metastatectomy	0.013		0.129
Yes		1	
No		2.37 (0.78-7.19)	
Number of metastatic organs	<0.001		0.037
Solitary		1	
Multiple		1.66 (1.03-2.66)	
Number of metastatic lesions	<0.001		0.004
≤3		1	
3<		2.07 (1.26-3.41)	
The largest diameter of metastatic foci	<0.001		0.007
≤5 cm		1	
5 cm<		2.75 (1.32-5.75)	
Bone metastasis	<0.001		0.106
No		1	
Yes		1.6 (0.9-2.89)	
Liver metastasis	<0.001		0.029
No		1	
Yes		2.27 (1.09-4.76)	
Lung metastasis	0.366		
No			
Yes			
Lymph node metastasis	0.403		
No			
Yes			

HR, hazard ratio; CI, confidence interval; PS, performance status; TC, total cystectomy; UC, urothelial carcinoma; CIS, carcinoma *in situ*; LVI, lymphovascular invasion

### Relationship between oligometastasis and clinicopathological features in the overall patient population

A total of 43 patients (33.6%) were in the OM group. The relationship between oligometastasis and clinicopathological characteristics in all patients is shown in Table 2. The performance status (PS) was better in the OM group than in the non-OM group. The size of metastatic foci was smaller and the number of metastatic organs and lesions were lower in the OM group than in the non-OM group. There was a lower population of patients with liver metastasis and lymph node metastasis in the OM group than in the non-OM group. Furthermore, the incidences of lymphovascular invasion (LVI) and the rate of neoadjuvant chemotherapy performed were lower in the OM group than in the non-OM group.

**Table 2:** Relationship between the oligometastatic status and clinicopathological features in all patients

	Oligometastasis group	Non-oligometastasis group	p value
	(n=43)	(n=85)	
Sex			0.425
Male	31 (72.1%)	64 (75.3%)	
Female	12 (27.9%)	21 (24.7%)	
Age at the diagnosis of metastasis			0.245
Mean±SD	68.3±9.64	70.4±9.76	
Time to metastasis from initial TUR-BT (month)			0.378
Mean±SD	40.0±143.9	68.3±215.0	
Time to metastasis from TC (month)			0.017
Mean±SD	11.1±13.6	17.7±15.9	
Previous history of smoking			0.566
Yes	21 (48.8%)	40 (47.1%)	
No	16 (37.2%)	31 (36.5%)	
Unknown	6 (14.0%)	14 (16.4%)	
PS at the diagnosis of metastasis			0.046
≤1	41 (95.3%)	71 (83.5%)	
2≤	2 (4.7%)	14 (16.5%)	
Number of metastatic organs			<0.001
Mean±SD	1.00±0.0	2.34±1.25	
Number of metastatic lesions			<0.001
Mean±SD	1.26±0.62	5.92±4.00	
Lymph node metastasis			0.006
Yes	16 (37.2%)	53 (62.4%)	
No	27 (62.8%)	32 (37.6%)	
Bone metastasis			0.186
Yes	6 (14.0%)	19 (22.4%)	
No	37 (86.0%)	66 (77.6%)	
Liver metastasis			0.006
Yes	0 (0.0%)	12 (14.0%)	
No	43 (100%)	73 (86.0%)	
Lung metastasis			0.404
Yes	9 (20.9%)	21 (24.7%)	
No	34 (79.1%)	64 (75.3%)	
The largest diameter of metastatic foci (cm)			0.001
Mean±SD (range)	2.35±1.26	3.34±1.71	
Clinical T stage			0.545
<cT2	9 (20.9%)	8 (9.4%)	
cT2	13 (30.2%)	33 (38.8%)	
cT3	15 (34.9%)	28 (32.9%)	
cT4	2 (4.7%)	6 (7.1%)	
Unknown	4 (9.3%)	10 (11.8%)	
Clinical N stage			0.436
cN0	33 (76.7%)	65 (76.5%)	
cN1≤	7 (16.3%)	17 (20.0%)	
Unknown	3 (7.0%)	3 (3.5%)	
Number of lymph nodes removed at TC			
Mean±SD (range)	12.8±8.23	12.1±8.23	
Pathological T stage on TC specimens			0.288
<pT2	9 (20.9%)	12 (14.0%)	
pT2	7 (16.3%)	14 (16.5%)	
pT3	22 (51.2%)	40 (47.1%)	
pT4	5 (11.6%)	19 (22.4%)	
Pathological N stage on TC specimens			0.256
pN0	33 (76.7%)	59 (69.4%)	
pN1≤	10 (23.3%)	26 (30.6%)	
Histological type			0.178
Pure UC	34 (79.1%)	74 (87.1%)	
UC with squamous cell carcinoma	4 (9.3%)	5 (5.8%)	
UC with adenocarcinoma	2 (4.7%)	6 (7.1%)	
UC with small cell carcinoma	2 (4.7%)	0 (0.0%)	
UC with micropapillary	1 (2.2%)	0 (0.0%)	
Concomitant CIS status on TC specimens			0.544
Yes	4 (9.3%)	9 (10.6%)	
No	39 (90.7%)	76 (89.4%)	
LVI status on TC specimens			0.009
Negative	21 (48.8%)	22 (25.9%)	
Positive	22 (51.2%)	63 (74.1%)	
Neoadjuvant chemotherapy			0.008
Yes	8 (18.6%)	35 (41.2%)	
No	35 (81.4%)	50 (58.8%)	
Adjuvant chemotherapy			0.417
Yes	10 (23.3%)	17 (20.0%)	
No	33 (76.7%)	68 (80.0%)	
Salvage chemotherapy			0.265
Yes	30 (69.8%)	53 (62.4%)	
No	13 (30.2%)	32 (37.6%)	
Metastatectomy			0.084
Yes	5 (11.6%)	3 (3.5%)	
No	38 (88.4%)	82 (96.5%)	

SD, standard deviation; PS, performance status; TC, total cystectomy; UC, urothelial carcinoma; CIS, carcinoma *in situ*; LVI, lymphovascular invasion.

### Prognostic significance of oligometastasis on overall death and cancer-specific death in the overall patient population

**Figure 1:**
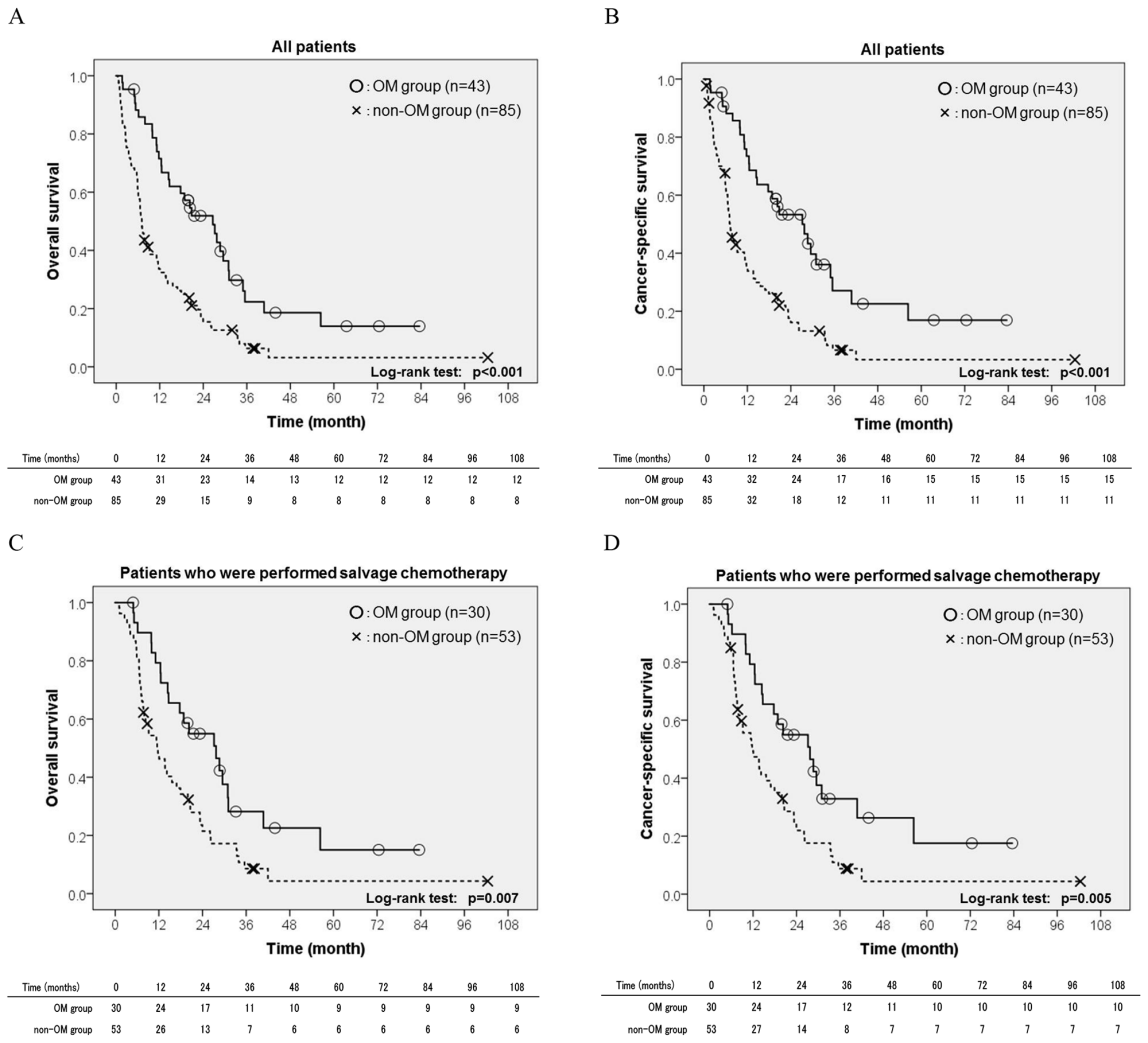
A Kaplan-Meier curve of overall survival in **(A)** the overall population and **(C)** patients who received salvage chemotherapy, and cancer-specific survival in **(B)** the overall population and **(D)** patients who received salvage chemotherapy according to the metastatic status (oligometastasis; OM group vs. non-oligometastasis; non-OM group).

Overall death developed in 108 patients (84.4%), comprising 31 patients in the OM group and 77 patients in the non-OM group. A Kaplan-Meier curve revealed that the rate of overall death was significantly lower in the OM group than in the non-OM group (p<0.001, Figure 1A). Two-year overall survival (OS) rates were 51.9% in the OM group and 15.4% in the non-OM group. The results of a univariate Cox regression analysis are shown in Table 3. A multivariate Cox regression analysis revealed that non-oligometastasis (hazard ratio: HR 2.96, p<0.001) was an independent risk factor for overall death in addition to not performing salvage chemotherapy (HR 3.02, p<0.001) and not performing metastatectomy (HR 3.91, p=0.02).

**Table 3:** Uni- and multivariate Cox regression analyses for overall and cancer-specific death according to clinicopathological features including oligometastatic status in 128 overall metastatic bladder cancer patients

	Overall death			Cancer-specific death		
	Univariate	Multivariate		Univariate	Multivariate	
	p value	HR (95% CI)	p value	p value	HR (95% CI)	p value
Sex	0.96			0.947		
Male						
Female						
Age at the diagnosis of metastasis	0.315			0.388		
<70						
70≤						
Time to metastasis from initial TUR-BT	0.053			0.095		
<50 months						
50 months≤						
Time to metastasis from TC	0.006		0.183	0.004		0.105
<12 months		1.36 (0.87-2.12)			1.46 (0.92-2.33)	
12 months≤		1			1	
Previous history of smoking	0.336			0.305		
Yes						
No						
PS at the diagnosis of metastasis	<0.001		0.127	0.001		0.214
≤1		1			1	
2≤		1.63 (0.87-3.07)			1.53 (0.78-2.99)	
Clinical T stage	0.392			0.352		
<cT3						
cT3≤						
Clinical N stage	0.084			0.094		
cN0						
cN1≤						
Number of lymph nodes removed	0.124			0.119		
<10						
10≤						
Pathological T stage	0.02		0.634	0.061		
<pT3		1				
pT3≤		1.36 (0.68-1.87)				
Pathological N stage	0.59			0.479		
pN0						
pN1≤						
Histological type	0.355			0.734		
Pure UC						
Non-pure UC						
Concomitant CIS	0.414			0.535		
Yes						
No						
LVI	0.004		0.696	0.004		0.572
Negative		1			1	
Positive		1.1 (0.68-1.8)			1.16 (0.69-1.91)	
Neoadjuvant chemotherapy	0.476			0.624		
Yes						
No						
Adjuvant chemotherapy	0.5			0.453		
Yes						
No						
Salvage chemotherapy	<0.001		<0.001	0.001		<0.001
Yes		1			1	
No		3.02 (1.89-4.85)			2.88 (1.77-4.69)	
Metastatectomy	0.008		0.02	0.013		0.028
Yes		1			1	
No		3.91 (1.23-12.3)			3.66 (1.15-11.8)	
Oligometastasis	<0.001		<0.001	<0.001		<0.001
Yes		1			1	
No		2.96 (1.86-4.69)			3.11 (1.92-5.03)	

HR, hazard ratio; CI, confidence interval; PS, performance status; UC, urothelial carcinoma; CIS, carcinoma *in situ*; LVI, lymphovascular invasion.

Cancer-specific death developed in 102 patients (79.7%), comprising 28 patients in the OM group and 74 in the non-OM group. A Kaplan-Meier curve revealed that the rate of cancer-specific death was significantly lower in the OM group than in the non-OM group (p<0.001, Figure 1B). Two-year cancer-specific survival (CSS) rates were 53.3% in the OM group and 16.1% in the non-OM group. The results of a univariate Cox regression analysis are shown in Table 3. A multivariate Cox regression analysis revealed that non-oligometastasis (HR 3.11, p<0.001) was an independent risk factor for cancer-specific death in addition to not performing salvage chemotherapy (HR 2.88, p<0.001) and not performing metastatectomy (HR 3.66, p=0.028).

The organs involved in oligometastasis were regional lymph nodes in 9, lung in 9, pelvis in 7, distant lymph nodes in 7, bone in 6, and ureter/renal pelvis in 5. No association between these organs involved in oligometastasis and cancer specific death was observed in the overall patients.

### Prognostic significance of oligometastasis on overall death and cancer-specific death in patients receiving salvage chemotherapy

A total of 30 patients (36.1%) were in the OM group. The relationship between oligometastasis and clinicopathological characteristics in patients receiving salvage chemotherapy is shown in Table 4. Among patients who received salvage chemotherapy, the OM group received neoadjuvant chemotherapy less frequently than the non-OM group. Furthermore, the size of metastatic foci was smaller and the number of metastatic organs and lesions were lower in the OM group than in the non-OM group. Overall death developed in 68 patients (81.9%), consisting of 21 patients in the OM group and 47 patients in the non-OM group. A Kaplan-Meier curve revealed that the rate of overall death was significantly lower in the OM group than in the non-OM group (p=0.007, Figure 1C). Two-year OS rates were 55.0% in the OM group and 21.5% in the non-OM group. The results of a univariate Cox regression analysis are shown in Table 5. A multivariate Cox regression analysis revealed that non-oligometastasis (HR 2.11, p=0.012) was the only independent risk factor for overall death in metastatic BC patients who received salvage chemotherapy.

**Table 4:** Relationship between the oligometastatic status and clinicopathological features in patients who received salvage chemotherapy

	Oligometastasis group	Non-oligometastasis group	p value
	(n=30)	(n=53)	
Sex			0.353
Male	24 (80.0%)	39 (73.6%)	
Female	6 (20.0%)	14 (26.4%)	
Age at the diagnosis of metastasis			0.742
Mean±SD	67.9±9.02	67.1±9.71	
Time to metastasis from initial TUR-BT (month)			0.517
Mean±SD	48.6±178.8	79.5±252.2	
Time to metastasis from TC (month)			0.112
Mean±SD	12.0±14.7	17.5±15.7	
Previous history of smoking			0.448
Yes	14 (46.6%)	22 (41.5%)	
No	11 (36.7%)	21 (39.6%)	
Unknown	5 (16.7%)	10 (18.9%)	
PS at the diagnosis of metastasis			0.255
≤1	30 (100%)	50 (94.3%)	
2≤	0 (0.0%)	3 (5.7%)	
Number of metastatic organs			<0.001
Mean±SD	1.00±0.0	2.17±1.19	
Number of metastatic lesions			<0.001
Mean±SD	1.33±0.71	5.89±3.79	
Lymph node metastasis			0.054
Yes	13 (43.3%)	34 (64.2%)	
No	17 (56.7%)	19 (35.8%)	
Bone metastasis			0.289
Yes	1 (3.3%)	5 (9.4%)	
No	29 (96.7%)	48 (90.6%)	
Liver metastasis			0.099
Yes	0 (0.0%)	5 (9.4%)	
No	30 (100%)	48 (90.6%)	
Lung metastasis			0.343
Yes	7 (23.3%)	16 (30.2%)	
No	23 (76.7%)	37 (69.8%)	
The largest diameter of metastatic foci (cm)			0.001
Mean±SD	2.07±1.16	3.19±1.64	
Clinical T stage			0.157
<cT2	6 (20.0%)	7 (13.2%)	
cT2	9 (30.0%)	24 (45.3%)	
cT3	12 (40.0%)	13 (24.5%)	
cT4	1 (3.3%)	4 (7.6%)	
Unknown	2 (6.7%)	5 (9.4%)	
Clinical N stage			0.5
cN0	23 (76.7%)	39 (73.6%)	
cN1≤	6 (20.0%)	12 (22.6%)	
Unknown	1 (3.3%)	2 (3.8%)	
Number of lymph nodes removed at TC			
Mean±SD (range)	11.5±7.27	12.5±8.44	
Pathological T stage on TC specimens			0.423
<pT2	6 (20.0%)	10 (18.9%)	
pT2	5 (16.7%)	12 (22.6%)	
pT3	14 (46.6%)	20 (37.7%)	
pT4	5 (16.7%)	11 (20.8%)	
Pathological N stage on TC specimens			0.574
pN0	23 (76.7%)	41 (77.4%)	
pN1≤	7 (23.3%)	12 (22.6%)	
Histological type			0.607
Pure UC	27 (90.0%)	48 (90.6%)	
UC with squamous cell carcinoma	1 (3.3%)	3 (5.7%)	
UC with adenocarcinoma	1 (3.3%)	2 (3.8%)	
UC with micropapillary	1 (3.3%)	0 (0.0%)	
Concomitant CIS status on TC specimens			0.522
Yes	4 (13.3%)	6 (11.3%)	
No	26 (86.7%)	47 (88.7%)	
LVI status on TC specimens			0.085
Negative	15 (50.0%)	17 (32.1%)	
Positive	15 (50.0%)	36 (67.9%)	
Neoadjuvant chemotherapy			0.001
Yes	5 (16.7%)	28 (52.8%)	
No	25 (83.3%)	25 (47.2%)	
Adjuvant chemotherapy			0.438
Yes	8 (26.7%)	12 (22.6%)	
No	22 (73.3%)	41 (77.4%)	
Metastatectomy			0.458
Yes	2 (6.7%)	2 (3.8%)	
No	28 (93.3%)	51 (96.2%)	

SD, standard deviation; PS, performance status; TC, total cystectomy; UC, urothelial carcinoma; CIS, carcinoma *in situ*; LVI, lymphovascular invasion.

**Table 5:** Uni- and multivariate Cox regression analyses for overall and cancer-specific death according to clinicopathological features including oligometastatic status in 83 metastatic bladder cancer patients who received salvage chemotherapy

	Overall death			Cancer-specific death		
	Univariate	Multivariate		Univariate	Multivariate	
	p value	HR (95% CI)	p value	p value	HR (95% CI)	p value
Sex	0.748			0.843		
Male						
Female						
Age at the diagnosis of metastasis	0.604			0.593		
<70						
70≤						
Time to metastasis from initial TUR-BT	0.078			0.098		
<50 months						
50 months≤						
Time to metastasis from TC	0.059			0.056		
<12 months						
12 months≤						
Previous history of smoking	0.167			0.211		
Yes						
No						
PS at the diagnosis of metastasis	0.033		0.287	0.029		0.268
≤1		1			1	
2≤		1.99 (0.56-7.02)			2.05 (0.58-7.26)	
Clinical T stage	0.572			0.636		
<cT3						
cT3≤						
Unknown						
Clinical N stage	0.199			0.15		
cN0						
cN1≤						
Unknown						
Pathological T stage	0.409			0.527		
<pT3						
pT3≤						
Pathological N stage	0.898			0.999		
pN0						
pN1≤						
Number of lymph nodes removed	0.288			0.224		
<10						
10≤						
Histological type	0.162			0.323		
Pure UC						
Non-pure UC						
Concomitant CIS	0.419			0.479		
Yes						
No						
LVI	0.057			0.09		
Negative						
Positive						
Neoadjuvant chemotherapy	0.756			0.796		
Yes						
No						
Adjuvant chemotherapy	0.25			0.323		
Yes						
No						
Metastatectomy	0.148			0.166		
Yes						
No						
Oligometastasis	0.007		0.012	0.005		0.009
Yes		1			1	
No		2.11 (1.18-3.77)			2.21 (1.22-4.01)	

HR, hazard ratio; CI, confidence interval; PS, performance status; CIS, carcinoma *in situ*; UC, urothelial carcinoma; LVI, lymphovascular invasion

Among patients who received salvage chemotherapy, cancer-specific death developed in 66 patients (79.5%), consisting of 20 in the OM group and 46 in the non-OM group. A Kaplan-Meier curve revealed that the rate of cancer-specific death was significantly lower in the OM group than in the non-OM group (p=0.005, Figure 1D). Two-year CSS rates were 55.0% in the OM group and 22.0% in the non-OM group, respectively. The results of a univariate Cox regression analysis are shown in Table 5. A multivariate Cox regression analysis revealed that non-oligometastasis (HR 2.21, p=0.009) was the only independent risk factor for cancer-specific death in metastatic BC patients who received salvage chemotherapy.

In 30 patients with oligometastatic BC who received salvage chemotherapy, 20 patients received GC therapy, 6 patients received MVAC therapy, and 4 patients received gemcitabine and paclitaxel (GP) therapy. The 2-year CSS rate in patients treated with GP therapy was 0%, which was significantly lower than that in those patients treated with GC therapy (62.7%, p<0.001) or MVAC therapy (66.7%, p=0.048).

## DISCUSSION

We retrospectively analyzed the records of patients who were diagnosed with metastatic BC after TC at our 6 institutions and investigated the relationship between prognoses and oligometastasis. Our results revealed that, in the overall population, non-oligometastasis, not performing salvage chemotherapy, and not performing metastatectomy were independent risk factors for overall death and cancer-specific death in metastatic BC after TC. In a subgroup of patients who received salvage chemotherapy, non-oligometastasis was the only independent predictor for overall death and cancer-specific death. Furthermore, we evaluated the association between oligometastatic organs and clinical outcome in the overall population. When different oligometastatic tissues were separately added as factors to univariate Cox regression analysis for cancer death, we found that none of these factors were identified as significant indicators for cancer death.

Oligometastasis was defined in 1995 as metastases limited in number and location because the facility for metastatic growth has not been fully developed and the site for growth is restricted [6]. Our study population only included oligometastases that occurred after surgery; therefore, in the narrow sense, this may be called oligorecurrence. The definition of oligometastasis differs somewhat among the types of cancers, but was generally created based upon the concept of “a solitary or a few detectable metastatic lesions of a small size that are generally confined to a single or a few organs”, according to previous studies [7, 12, 13]. Therefore, the metastatic site, number, and tumor diameter are particularly important for establishing a definition for oligometastasis. In the present study, we used criteria that included these important factors based on previous findings.

Reyes et al. described the biology of oligometastatic disease [7]. The metastatic growth potential of oligometastatic disease is limited and this may be secondary to environmental conditions in the primary tumor forestalling evolutionary clonal pressure. Although cancer cells slough out of the primary tumor and land at target organ sites, they do not have the properties necessary to survive the circulation and invade target organ sites. Oligometastatic foci generally have the same properties as tumor cells derived from the primary organ that are primarily sensitive to systemic chemotherapy. Therefore, in oligometastatic disease, the efficacy of systemic chemotherapy may be favorable. Greenberg et al. previously conducted a long-term follow-up of patients with complete remission following chemotherapy for metastatic breast cancer and showed that so-called oligometastasis may be more sensitive to systemic chemotherapy than widely disseminated metastatic disease [14]. They found that the number of metastatic sites was lower in patients with long-term complete remission than that in those with short-term complete remission. We speculate that differences in biological potential such as microRNA expression levels are associated with responses to systemic chemotherapy. MicroRNA profiling may be used to distinguish patients with oligometastasis from those with polymetastatic disease. Lussier et al. reported that microRNA-200c and 328 expression were significantly higher in oligometastasis than in non-oligometastasis [15]. Furthermore, the over or under expression of microRNAs may play a crucial role in the acquisition of chemo-resistance by cancer cells [16-18]. Several investigators showed that the overexpression of microRNA-155, 222, 125b, and 21 and the decreased expression of microRNA-200c and 328 correlated with a good response to chemotherapy. Further studies are needed in order to confirm the relationship between the expression levels of these microRNAs and chemosensitivity in UC.

The concept of oligometastasis has also been introduced for types of cancers with relatively unfavorable prognoses such as metastatic soft tissue sarcoma [19, 20] as well as metastatic melanoma [21]. In the field of metastatic UC, Abe et al. investigated the prognostic factors of metastatic UC treated by systemic chemotherapy and reported that single organ metastasis was identified as a favorable prognostic factor in addition to being female, having a good PS, hemoglobin level ≥10 g/dl, and undergoing metastasectomy [22]. However, the relationship between oligometastasis and prognoses in metastatic UC has not yet been examined. Our results showed a clear relationship between oligometastasis and a good prognosis in metastatic BC patients not only in the overall population, but also in a subgroup receiving salvage chemotherapy. If the concept of oligometastasis in metastatic BC is accepted, we may extract a group of patients with a better prognosis and chemosensitivity, the “oligometastasis group”, from a heterogeneous population with metastatic BC. As a result, we may construct risk stratification and create a management strategy according to the oligo- or non-oligo-metastatic status. The concept of “oligometastasis” may also provide useful counseling information for metastatic BC patients.

There were several limitations to the present study. It was performed in a retrospective manner and, thus, unknown sources of bias may exist in the results obtained. Whether salvage chemotherapy was performed is one of our selection biases. Performing salvage chemotherapy was selected based on the patients’ desire or physicians’ preference. In the present study, only 65% of metastatic BC patients received salvage chemotherapy, whereas in real clinical settings, not all metastatic BC patients receive systemic chemotherapy [23]. Furthermore, our study only included patients with metastatic BC after undergoing TC. Therefore, it was not possible to evaluate oligometastasis and the prognosis of patients initially diagnosed with metastatic BC at presentation. A larger study is warranted in order to clarify the relationship between oligometastasis and prognoses in initially diagnosed and post-operative recurrent/metastatic BC patients.

## MATERIALS AND METHODS

### Patient selection

We retrospectively reviewed medical records between 2004 and 2014 archived at our 6 institutions. In the present study, 506 consecutive patients underwent TC. After surgery, patients were generally followed-up at least every 3-4 months for 2 years, then every 6 months until 5 years, and annually thereafter. Follow-up visits consisted of a physical examination and serum routine blood tests. Diagnostic imaging including computed tomography of the chest/abdomen/pelvis with or without intravenous contrast was performed every 6 months until 5 years and then annually or when clinically indicated. GC, MVAC, or a GP chemo-regimen was used in neoadjuvant and/or salvage settings. We used different chemo-regimens in salvage settings from those in neoadjuvant settings if metastasis occurred within 1 year of TC, and if metastasis occurred more than 1 year after TC, the same chemo-regimen in salvage settings as those in neoadjuvant settings was selected.

A total of 149 patients had metastatic lesions after TC. Disease recurrence was defined as any documented recurrence by radiographically or pathologically proven failure in local and distant sites. We excluded 11 patients who were data deficiency or had a short observation period (less than 1 year) and 10 patients who were not diagnosed with UC. After the exclusion of these patients, the remaining 128 patients were assessed in the present study.

### The analysis of the independent predictors for identifying a favorable prognosis

We initially extracted independent predictors for identifying a favorable prognosis in patients with metastatic BC after TC using a multivariate Cox regression analysis. We repeated multivariate analyses using various cut-off values for the number of metastatic organs, number of metastatic lesions, the largest diameter in metastatic foci, and metastatic organ sites.

### Statistical analysis

The relationships between clinicopathological features and oligometastasis were analyzed using the χ^2^ test or Mann–Whitney U test. OS was defined as the time from the diagnosis of metastasis until death (all causes). CSS was defined as the time from the diagnosis of metastasis until death by BC. OS and CSS rates were estimated using Kaplan-Meier curves and compared using the Log-rank test. Independent variables included in the present study were sex, patient age (<70 vs. 70≤ years), a previous history of smoking, PS (≤1 vs. 2≤), clinical T stage (<T3 vs. T3≤), clinical N stage (N0 vs. N1≤), pathological T stage (<T3 vs. T3≤), pathological N stage (N0 vs. N1≤), number of resected lymph nodes (<10 vs. 10≤), histological type (pure UC vs. non-pure UC), presence or absence of concomitant carcinoma *in situ* (CIS), positive or negative LVI, whether neoadjuvant chemotherapy was performed, whether adjuvant chemotherapy was performed, whether salvage chemotherapy was performed, whether metastatectomy was performed, and oligometastatic status. A multivariate analysis was performed using Cox’s proportional hazard model with a stepwise forward selection method. Differences among the 2 groups were considered to be significant at p<0.05. These analyses were performed with the SPSS v. 22.0 statistical software package (IBM Corp., Somers, NY).

## CONCLUSIONS

Metastatic BC patients after undergoing TC with oligometastasis and/or receiving salvage chemotherapy had better prognoses. In the subgroup of patients who received salvage chemotherapy, oligometastasis was the only independent prognostic factor for cancer death. Urologists need to introduce the concept of “oligometastasis” for the prediction of prognoses and management of metastatic BC.

## Abbreviations

BC: bladder cancer
OM group: oligometastasis group
Non-OM group: non-oligometastasis group
CSS: cancer-specific survival
MVAC: methotrexate, vinblastine, doxorubicin, and cisplatin
GC: gemcitabine and cisplatin
GP: gemcitabine and paclitaxel
UC: urothelial carcinoma
TC: total cystectomy
OS: overall survival
PS: performance status
CIS: carcinoma *in situ*
LVI: lymphovascular invasion
HR: hazard ratio
SD: standard deviation
CI: confidence interval
